# Impact of mushroom (*Pleurotus eryngii*) flour upon quality attributes of wheat dough and functional cookies‐baked products

**DOI:** 10.1002/fsn3.1315

**Published:** 2019-12-25

**Authors:** Yuan Biao, Xin Chen, Song Wang, Guitang Chen, David Julian Mcclements, Liyan Zhao

**Affiliations:** ^1^ College of Food Science and Technology Nanjing Agricultural University Nanjing China; ^2^ Department of Food Quality and Safety/National R&D Center for Chinese Herbal Medicine Processing College of Engineering China Pharmaceutical University Nanjing China; ^3^ Department of Food Science University of Massachusetts Amherst MA USA

**Keywords:** cookies, dough, mushroom, orthogonal test, rheological properties

## Abstract

The objective of this study was to create a healthier version of a commonly consumed baked food (cookies) by replacing some of the wheat flour with a nutraceutical‐rich mushroom flour. The impact of incorporating different levels of powdered mushroom (*Pleurotus eryngii*) flour on the rheological properties of the cookie dough and the final cookies was therefore determined. The rheological properties of wheat dough supplemented with 0%–25% (*w*/*w*) of mushroom flour were analyzed using a Mixolab instrument and a shear rheometer. Increasing the ratio of mushroom‐to‐wheat flour in the doughs increased the peak and final viscosities, but decreased dough stability and elastic modulus. Sensory evaluation using an orthogonal test showed that 15% mushroom flour, 10% maltodextrin, and 1.5% sodium bicarbonate were the optimal composition for producing cookies with the best sensory score. In conclusion, our results showed that cookies with acceptable textures and appearances could be produced by replacing up to 15% of wheat flour with mushroom flour.

## Graphical abstract

Edible mushrooms are widely appreciated for their nutritional, bioactive activities and therapeutic potential. The enrichment of cookies with mushroom (*Pleurotus eryngii*) flour would be achievable and valuable to provide people with nutraceutical‐rich product in their daily diet. It would be important that his study indicated that incorporation less than 15% *P. eryngii* flour had acceptable formation, texture, and appearance of cookies.

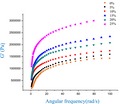

## 1 INTRODUCTION

Edible mushrooms are traditionally consumed for their desirable textures and flavors. Recent research, however, has focused on the fact that they also contain a variety of bioactive components that can also enhance the health benefits of foods (Aida, Shuhaimi, Yazid, & Maaruf, 2009). Consequently, there is considerable interest in using mushrooms as functional ingredients that can improve the nutritional profile of foods, such as breads, cookies, and meats (Bao, Ushio, & Ohshima, 2009; Lee & Jeong, 2009; Yuan, Zhao, Yang, McClements, & Hu, 2017). However, mushroom addition should not adversely affect the desirable appearance, texture, flavor, and shelf life of functional foods. *Pleurotus eryngii* is one of the most widely consumed mushrooms in many Asian countries (Aida et al., 2009; Yuan, Ma, et al., 2017). It is a natural source of bioactive compounds, including carbohydrates, peptides, dietary fibers, and nutraceuticals (Yuan, Ma, et al., 2017; Yuan, Zhao, Rakariyatham, et al., 2017). Indeed, studies have shown that *P. eryngii* exhibits good antioxidant, anti‐inflammatory, anticolon cancer, and anticolitis properties (Hu et al., 2019; Yuan, Ma, et al., 2017). For this reason, there has been considerable interest in incorporating *P. eryngii* into foods, such as baked goods, to improve their nutritional profiles and potential health benefits.

In this study, we examined the possibility of improving the health profile of a commonly consumed baked good (cookies) by replacing some of the wheat flour with nutraceutical‐rich mushroom flour. Cookies contain three major calorific components (flour, sugar, and fat) and a small amount of water. Flour, sugar, fat, and water are mixed together with some minor components to form dough (Zucco, Borsuk, & Arntfield, 2011). Dough formation, cookie baking, and cookies quality are strongly affected by ingredient properties (Hasmadi & Sandra, 2014; Laura, Ana, Teresa, & Susana, 2011; Pareyt & Delcour, 2008).

A few researchers have previously examined the impact of incorporating mushroom flours into breads and cookies on their physicochemical and sensory properties (Jung & Joo, 2010; Lee & Jeong, 2009; Raimondo et al., 2018; Ulziijargal, Yang, Lin, Chen, & Mau, 2013; Yuan, Zhao, Yang, et al., 2017). These studies all suggested that mushroom flour could be successfully incorporated into baked goods without adversely affecting their preparation and final properties, provided too much was not added. Thus, fortification of cookies with milled mushroom flour could be a good approach to enhance the nutritional value of these baked goods. However, there is currently little knowledge about the influence of *P. eryngii* flour on the formation and quality attributes of cookies, especially on the rheological properties of cookie dough formulations. Thus, the present study was designed to evaluate the impact of partial replacement of wheat flour with mushroom (*P. eryngii*) flour on cookie manufacture, as well as on the quality of the dough and cookies produced. In particular, the study aimed to evaluate the effects of adding different levels of milled mushroom flour (5%, 10%, 15%, or 20%) on the rheological properties of doughs, as well as to optimize the levels of mushroom flour, maltodextrin, and sodium bicarbonate required to produce cookies with desirable sensory attributes.

## 2 MATERIALS AND METHODS

### 2.1 Materials

Mushrooms (*P. eryngii*) were bought from a supermarket. They were then frozen at −20°C and freeze‐dried using a lab‐scale freeze dryer (Labconco Equipment Co.) at 40°C for 8 hr with chamber pressure of 100 Pa and condenser temperature of −84°C as previously reported (Pei et al., 2014). After that, the freeze‐dried mushrooms were ground into a fine powder (80‐mesh, 0.2 mm) using a laboratory grinder (IKA‐Werke, M20) and stored at −80°C for further analysis (Moreira, Chenlo, Torres, & Prieto, 2010). Cookie flour, sugar, salt, edible oil, sodium bicarbonate, and maltodextrin were purchased from a supermarket. All other chemicals used were of analytical grade.

### 2.2 Compositional analysis

Proximate analyses of mushroom and wheat flour, including moisture, total crude protein, lipid content, and ash content, were performed using standard methods: Methods 925.10, 979.09, 963.15, and 923.03, respectively (Association of Official Analytical Chemists, 2000). Total dietary fiber content was measured by a commercial total dietary fiber assay kit (AOAC 985.29) (St. Louis, Missouri, USA). Total starch content was measured by a commercial Megazyme Total Starch kit (AA/AMG, Megazyme Pty Ltd.).

### 2.3 Pasting properties

Rapid Visco Analyser (RVA) analysis was carried out to determine the pasting properties of the flour suspensions (Model Super 3, Newport Scientific) as described previously. Suspensions were prepared by mixing deionized water (25 ml) with flour (3.5 g) and then manually homogenizing using a plastic paddle. Tests were conducted using a programmed heating and cooling method: holding at 50°C for 1 min; heat at 12°C/min to 95°C; holding at 95°C for 2.5 min; cool at 12°C/min to 50°C (3.7 min); hold at 50°C for 1.5 min. The plastic paddle was kept for 10 s at a speed of 1,000 *g*, and the rest time was kept at a speed of 200 *g*.

### 2.4 Thermo‐mechanical behavior

A Mixolab instrument (Chopin Technologies) was used to determine the thermo‐mechanical properties. This device measures dough consistency during the mixing procedure of controlled heating and cooling (Serpil, Kevser, Bengihan, & Hamit, 2008; Yuan, Zhao, Yang, et al., 2017). The “Chopin+” protocol was used during the measurement (ICC, 2006). Sufficient water was added to produce a “C1” consistency torque of 1.1 Nm (Rosell, Collar, & Haros, 2007). The program settings were as following: holding at 30°C for 8 min; heating at 4°C/min to 90°C; holding at 90°C for 7 min; cooling at 4°C/min to 50°C; holding for 5 min. The total time for this procedure was 45 min. The parameters measured included: the *minimum torque* produced by the dough related to protein weakening (C2 value); the *maximum torque* during the heating stage (C3 value); the *minimum torque* during the heating stage (C4 value); the *torque after cooling* to 50°C (C5 value); *dough development time* (DT); and *dough stability time* (ST). The control group refers to dough samples without mushroom flour added.

### 2.5 Oscillatory shear rheological measurement

Doughs with target “C1” consistency measured using the Mixolab were used in the rheological studies (Maria, Maria, Alberto, & Pablo, 2017). Dough oscillatory shear rheological properties were determined using a controlled‐stress rheometer (MCR 301, Anton Paar Physica). The measuring system contained a parallel plate geometry with the diameter of 50‐mm diameter and a gap of 2 mm. Dough samples taken from the innermost parts were loaded between two parallel plates. The rim of the sample was coated with paraffin to prevent evaporation while the measurements were being taken. The model of frequency sweep tests was conducted at 30°C from 0.01 to 10.00 Hz to measure the storage modulus and loss modulus.

### 2.6 Cookie preparation

The process adopted for preparation of cookies included slight modification in AACC standard method (10‐50D). A convection Model TRTF32A oven (Wei Shida Electrical Industrial Co., Ltd.) was used for the cookies bake. A T‐type thermocouple was used in the oven to monitor the temperature during the baking period. Then, the dough was kneaded and sheeted on a dough sheeter to obtain a uniform thickness of 0.5 cm and then cut into round shapes of 5 cm in diameter. The cookie was placed and baked at the temperature of 205°C for 10 min in the center of the oven. After that, the weights of the six cookies and cookie sheet were recorded. Then, the cookies were cooled at room temperature for further analysis. Overall, the baking cookies were replicated six times (Mehri & Francis, 2009).

### 2.7 Texture analysis

Cookies firmness was conducted using a TA‐XT2 Texture Analyzer compression machine (Stable Micro Systems) equipped with a HDP/3PB three‐point bending rig according to the established method (AACC‐10‐50D). Setting procedure on texture analyzer of freshly cooled cookies was as follows: pretest speed of 1.0 mm/s, a test speed of 3 mm/s, and a post‐test speed of 10.0 mm/s with a 10‐mm distance. Six measurements were conducted for each sample.

### 2.8 Orthogonal experimental design

#### 2.8.1 Effect of the addition of mushroom flour

The formulas of cookie samples preparation were 40% distilled water, 5% sugar, 20% maltodextrin, 0%, 5%, 10%, 15%, or 20% mushroom flour, with the remainder being wheat flour (wt% on 100 g basis).

#### 2.8.2 Effect of the addition of maltodextrin

The formulas of cookie samples preparation were 40% distilled water, 5% sugar, and 0%, 5%, 10%, 15%, 20%, 25%, or 30% maltodextrin, with the remainder being wheat/mushroom flour (wt% on 100 g basis).

#### 2.8.3 Effect of addition of NaHCO_3_

The formulas of cookie samples preparation were 40% distilled water, 5% sugar, and 20% maltodextrin, 0%, 0.5%, 1.0%, 1.5%, 2.0%, 2.5% or 3.0% sodium bicarbonate, with the remainder being wheat/mushroom flour (wt% on 100 g basis).

#### 2.8.4 Orthogonal experimental design

Based on single‐factor experiments, an orthogonal experiment was designed. Addition of mushroom flour (A), addition of maltodextrin (B), and addition of NaHCO_3_ (C) were the three main factors affecting the quality of the product. Consequently, we carried out an L_9_ (3)^4^ orthogonal experiment.

#### 2.8.5 Sensory evaluation

The sensory evaluation of the cookies was conducted in the sensory evaluation room in the College of Food Science and Technology in Nanjing Agricultural University. The sensory scores were evaluated by a panel of 20 semitrained people (Amerine, Pangborn, & Roessler, 1965). Panelists were trained for descriptive analysis for cookies sensory evaluation. Then, they were asked to evaluate the color of cookies first and then to evaluate their mouthfeel, texture, and flavor. Freshly made cookies were served on plates with random three‐digit codes to prevent any potential bias. The overall acceptability of cookies was calculated from the average values of all above sensory parameters. Panelists rinsed mouth with water between sample evaluations. A nine‐point hedonic scale was used for sensory evaluation.

### 2.9 Statistical analysis

All results are presented as means ± standard deviation (*SD*) of at least 3 repeated experiments. We used one‐way Analysis of Variance and Tukey's test (*p*‐value of <.05) to evaluate the significant differences between different groups using statistical software (PASW, SPSS Inc., Chicago, USA).

## 3 RESULTS AND DISCUSSION

### 3.1 Proximate component analysis

The mushroom flour contained 8.5% moisture, 19.1% crude protein, 1.3% fat, 8.6% total fiber, 5.6% ash, and 65.4% total carbohydrates. The wheat flour contained 12.3% moisture, 8.0% crude protein, 0.7% fat, 2.8% total fiber, 0.5% ash, and 78.5% total carbohydrates. The starch contents of the mushroom and wheat flours were 42.8% and 66.6%, respectively. The mushroom flour had more than fivefold higher levels of dietary fiber and minerals than that of the wheat flour, making it a better source of these beneficial food constituents. Supplementation of wheat flour with mushroom flour would therefore improve the nutritional profile of cookies.

### 3.2 Pasting properties

The pasting properties of flours are particularly important in the cookie‐making process, and therefore, the impact of blending the wheat flour with different levels of mushroom flour was examined. The paste viscosity then increased progressively as the samples were cooled, which can be attributed to a coil‐to‐helix transition and hydrogen bond formation between amylose molecules. The paste viscosity profile of samples containing different levels of mushroom flour was analyzed and various parameters determined (Table 1). The peak viscosities obtained with blended flours containing more than 5% (*w/w*) mushroom flour were significantly higher than those for wheat flour alone (*p* < .05).

**Table 1:** Effect of incorporation of mushroom flour on the pasting properties of wheat flour

Mushroom flour levels (w/w)	Peak visc (cP)	Holding visc (cP)	Breakdown (cP)	Final visc (cP)	Setback (cP)	Peak time (min)	Pasting temp (°C)
0%	2,600 ± 78a	1,618 ± 88a	983 ± 38a	2,928 ± 65a	1,310 ± 25a	6.07 ± 0.19a	67.05 ± 2.30b
5%	2,378 ± 96ab	1,366 ± 76ab	1,012 ± 58a	2,340 ± 47b	1,111 ± 23ab	5.80 ± 0.13b	84.05 ± 1.56a
10%	2,327 ± 36b	1,318 ± 32bc	1,009 ± 31a	2,255 ± 33bc	936 ± 77abc	5.80 ± 0.16b	85.60 ± 1.30a
15%	2,188 ± 45b	1,223 ± 34bc	965 ± 46a	2,149 ± 43bc	927 ± 38bc	5.80 ± 0.11b	85.55 ± 0.67a
20%	2,061 ± 47c	1,171 ± 23bc	891 ± 39ab	2,040 ± 23cd	869 ± 35c	5.67 ± 0.09c	85.35 ± 0.78a
25%	1,962 ± 28c	1,092 ± 67c	857 ± 32b	1,893 ± 54d	802 ± 25c	5.73 ± 0.02bc	85.50 ± 1.20a

Note

Breakdown = Peak Viscosity–Holding Viscosity; Setback = Final Viscosity–Peak Viscosity; Total Setback = Final Viscosity–Holding Viscosity. Each value is expressed as mean ± standard deviation (*n* = 3). Values with different superscript letters within a column are significantly different (*p* < .05).

As the proportion of mushroom flour increased, the peak and holding viscosities of the blended flours decreased. The presence of high levels of mushroom flour has a dilution effect on the content of wheat gluten in the blended flours, which leads to a decrease in dough stability. The final viscosity decreased from around 2,930 cP for wheat flour alone to 2,149 cP for the blended flour containing 15% (w/w) mushroom flour (*p* < .01; Table 1). The setback viscosity represents the difference between the final viscosity and the holding viscosity and describes the increase in viscosity that occurs when the starch paste cools down. The setback viscosity of the blended flour decreased as the amount of mushroom flour present was increased (Table 1), which may again be attributed to the diluting properties of the mushroom flour in the blended flour.

### 3.3 Thermo‐mechanical properties

Dough development time (DT) is highly related to water absorption capacity of dough and the gluten matrix strength (Rosell et al., 2007). Earlier studies have reported that replacing wheat flour with up to 6% (w/w) *Lentinus edode* flour or up to 7.5% (w/w) *Auricularia auricular* flour had little impact on dough development time (Heo et al., 2013; Yuan, Zhao, Yang, et al., 2017). In our study, replacing wheat dough with more than 10% mushroom flour led to a reduction in the dough development time in a dose dependent manner. This may be the result of faster absorption of water by the mushroom flour. The mushroom flour has a higher fiber content than the wheat flour. As a result, it may have adsorbed more water through capillary forces and hydrogen bonding. Moreover, the entanglement of the fibers would increase the resistance of the dough to flow, thereby increasing its yield stress and apparent viscosity (Ilkem, 2016; Ilkem, Behic, Gulum, & Serpil, 2010). Doughs with less than 10% mushroom flour had longer development times (3 min) compared with those containing more than 10% mushroom flour (<3 min). This effect may have been because the higher fiber content of the mushroom flour led to more water binding, thereby reducing the amount of water available to exhibit plasticizing effects (Nelson, 2001).

The dough stability time in the control group was 6.81 min, while that of the dough in the dough supplemented with 25% mushroom flour decreased to 4.13 min (*p* < .01; Table 2). This could be the consequence of higher amylolytic activity and/or changes in the morphology of the starch granules. The breakdown values decreased as the level of mushroom flour in the dough was increased, which indicates weaker dough stability. During the kneading period, the mechanical strength of the dough may have increased due to the formation of a three‐dimensional network of aggregated gluten. Adding mushroom flour into the dough reduces the total content of gluten in the blended system, thereby decreasing the dough stability time during kneading (Brennan, Derbyshire, Tiwari, & Brennan, 2012; Yuan, Zhao, Yang, et al., 2017). Previously, the incorporation of cowpea or buckwheat flours into wheat flour has been reported to reduce the mechanical strength of dough during the kneading period, which was attributed to a decrease in the total gluten content of the system (Hallén, İbanoğlu, & Ainsworth, 2004; Sedej et al., 2011).

**Table 2:** Effect of incorporation of mushroom flour on the thermo‐mechanical properties of wheat flour

*Pleurotus eryngii* levels (w/w)	WA (%)†	DT (min)	ST (min)	C2	C3	C4	C5
0%	54.81 ± 0.32f	3.62 ± 0.32a	6.81 ± 0.42a	0.44 ± 0.01a	2.01 ± 0.12a	1.85 ± 0.12a	2.07 ± 0.04a
5%	56.0 ± 0.43e	3.01 ± 0.21c	4.65 ± 0.32b	0.39 ± 0.00b	1.93 ± 0.12b	1.80 ± 0.0.3a	1.78 ± 0.13b
10%	57.5 ± 0.11d	3.22 ± 0.13b	4.32 ± 0.12bc	0.33 ± 0.01c	1.80 ± 0.16c	1.67 ± 0.0.7b	1.47 ± 0.22c
15%	59.6 ± 0.23c	2.58 ± 0.23d	3.78 ± 0.25d	0.31 ± 0.01d	1.75 ± 0.21d	1.64 ± 0.09b	1.21 ± 0.13d
20%	61.5 ± 0.32b	2.55 ± 0.17d	4.11 ± 0.11c	0.28 ± 0.01d	1.72 ± 0.17e	1.60 ± 0.07b	1.18 ± 0.08d
25%	62.6 ± 0.43a	1.98 ± 0.14e	4.13 ± 0.23c	0.28 ± 0.02d	1.63 ± 0.31f	1.54 ± 0.11b	0.97 ± 0.03e

Note

Data are expressed as mean ± standard deviation (*n* = 3). Values with different superscript letters within a row are significantly different (*p* < .05).

^†^ WA, water absorption level. C2, minimum torque produced by dough subjected to mechanical and thermal constraints; C3, maximum torque produced during heating stage; C4, minimum torque during heating period; C5, the torque obtained after cooling at 50°C; DT, dough development time (time required to reach C1 (in this case, 1.1 ± 0.07 Nm)).

The C2 values provide information about the extent of gluten network weakening during dough kneading: the higher the C2 value, the lower the degree of protein weakening (Ajila, Leelavathi, & Rao, 2008; Rosell, Santos, & Collar, 2010). In our study, the C2 value increased from 0.28 Nm in the control to 0.44 Nm in the dough containing 25% mushroom flour, suggesting a lower degree of gluten network weakening during kneading. The C3 value provides information about the degree of starch gelatinization during the dough kneading period (Wang, Rosell, & Benedito de Barber, 2002). As the addition of mushroom flour increased, the C3 values of the blended doughs and the starch gelatinization degree decreased significantly (*p* < .05).

The C4 and C5 values decreased significantly with increasing mushroom flour content in the blended dough (Table 2). Starch retrogradation could reduce the shelf life of cookies and adversely affects the textural and sensory properties of cookies (Smith, Daifas, El‐Khoury, Koukoutsis, & El‐Khoury, 2004). Therefore, the ability of mushroom flour to decrease the retrogradation degree may have beneficial role to cookie quality and shelf life. Also, lower C4 and C5 values were reported previously to have a good correlation with reduced staling of breads supplementing with other grain flours (Jia, Huang, Abdel‐Samie, Huang, & Huang, 2011).

### 3.4 Dough rheological properties

**Figure 1:**
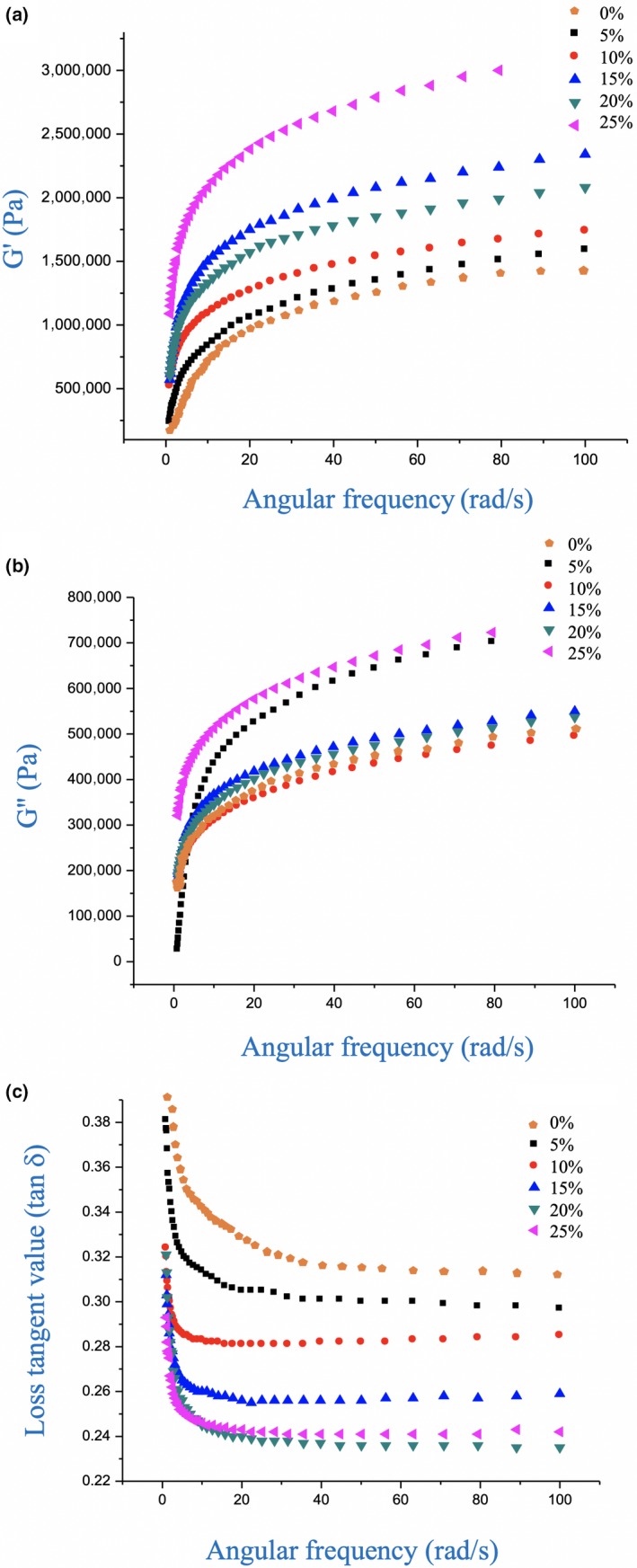
Effect of *Pleurotus eryngii* flour on dough dynamic viscoelastic properties by frequency sweep. (a) Storage modulus, *G*′, (b) loss modulus, *G*″, (c) loss tangent, tan *δ*

The dough rheological properties were characterized using isothermal oscillatory shear measurements to gain insight into the effect of mushroom flour supplementation on their rheological behavior. For all samples, when the angular frequency (*ω*) increased, the storage modulus (*G*′) and loss modulus (*G*″) of dough increased, with *G*′ > *G*″ at all *ω* values (Figure 1), indicating that the elastic properties of the doughs dominated their viscous properties (Khoigani, Rajaei, & Goli, 2017). At 20% and 25% mushroom flour, the *G*′ values of the doughs were higher than those of the control. Thus, the viscoelastic properties of the dough were also dependent on the mushroom flour content. This may have been because the entanglement of the fibers in the mushroom flour led to an increase in the elastic modulus of the dough samples. In addition, the fibers in the mushroom flour may have interacted with the gluten protein and strengthened the dough structure, leading to a higher elastic modulus. An earlier study reported similar findings, with the *G*′ values increasing when chestnut flour was incorporated into wheat dough (Moreira, Chenlo, & Torres, 2013).

### 3.5 Cookies properties

**Figure 2:**
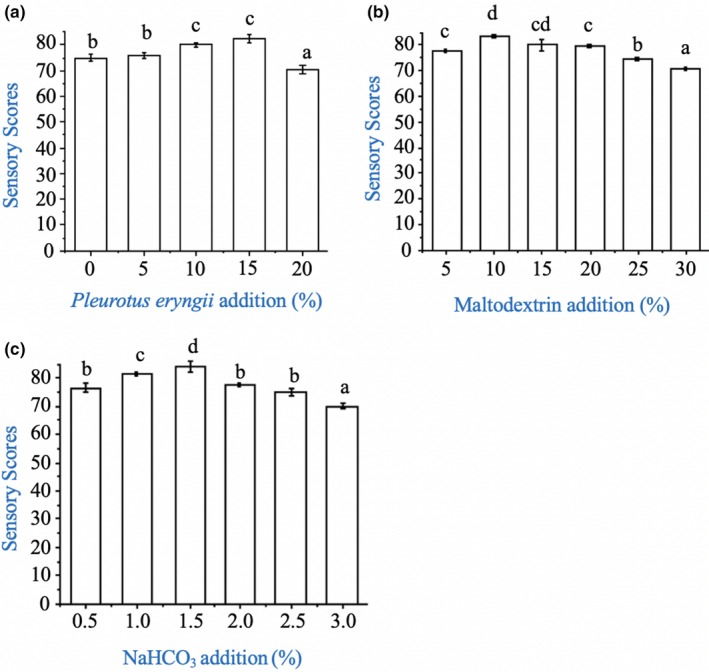
Effect of *Pleurotus eryngii* flour addition (a), maltodextrin addition (b), NaHCO_3_ addition (c) on the sensory scores (overall acceptability) of cookies

In these experiments, the impact of maltodextrin, sodium bicarbonate, and mushroom flour on the properties of the cookies was determined. Initially, the effect of maltodextrin addition on the sensory scores of the cookies was measured (Figure 2b). The sensory scores of the cookies improved when the level of maltodextrin added was increased from 5% to 10%, but then went down when more maltodextrin was added. The effect of adding sodium bicarbonate on the sensory attributes of the cookies was also determined (Figure 2c). The sensory attributes of the cookies increased when the NaHCO_3_ content was increased from 0.5% to 1.5%, but then decreased when it was further increased to 3.0%, which might have been because it led to a bitter taste.

The effect of three factors (A: addition of mushroom flour, B: addition of maltodextrin, and C: addition of NaHCO_3_) on the hardness, brittleness, and sensory evaluation scores of cookies was investigated (Table 3). Within a certain range, the hardness and brittleness positively related to the quality of the cookies, as shown in Table 4. The values of *k* and *R* were calculated using orthogonal analysis. According to the *R* values, the factors influencing the cookie hardness were as follows: A > C > B. The fact that the B‐factor (Addition of maltodextrin) was smaller than the D‐factor (Blank) suggested that maltodextrin addition had little influence on the hardness of the cookies. According to the *R* values, the factors influencing the brittleness were as follows: A > B > C.

**Table 3:** Design of orthogonal experiment

Levels	Addition of *Pleurotus eryngii* (%)	Addition of maltodextrin (%)	Addition of NaHCO_3_ (%)
1	0	0	0
2	5	5	0.5
3	10	10	1.0
4	15	15	1.5
5	20	20	2.0
6		25	2.5
7		30	3.0

**Table 4:** Effect of mushroom flour on the physical properties of cookies (L_9_(3)^4^ results)

No.	(A)	(B)	(C)	(D)	Hardness	Brittleness	Overall acceptability
	Addition of *Pleurotus eryngii* (%)	Addition of maltodextrin (%)	Addition of NaHCO_3_ (%)	Blank			
1	1 (10%)	1 (5%)	1 (1%)	1	1,978 ± 33d†	930 ± 8a	71 ± 1e
2	1	2 (10%)	2 (1.5%)	2	2,046 ± 23c	919 ± 3c	79 ± 1c
3	1	3 (15%)	3 (2%)	3	2,075 ± 25c	926 ± 4b	75 ± 1d
4	2 (15%)	1	2	3	1,948 ± 13d	939 ± 3a	83 ± 1b
5	2	2	3	1	2,105 ± 14b	903 ± 3d	85 ± 2a
6	2	3	1	2	2,170 ± 17a	843 ± 11e	89 ± 2a
7	3 (20%)	1	3	2	1,801 ± 36e	801 ± 12f	68 ± 3f
8	3	2	1	3	1,753 ± 43f	834 ± 9e	71 ± 1e
9	3	3	2	1	1,649 ± 25g	804 ± 15f	70 ± 1e
*k*_1_ (Hardness)	2,033	1,909	1,967	1,911			
*k*_2_ (Hardness)	2,074	1,968	1,881	2,006			
*k*_3_ (Hardness)	1,734	1,965	1,994	1,925			
*k*_1_ (Brittleness)	925	890	869	879			
*k*_2_ (Brittleness)	895	885	887	854			
*k*_3_ (Brittleness)	813	858	877	900			
*k*_1 _(Sensory quality)	75.0	74.0	77.0	75.3			
*k*_2_ (Sensory quality)	85.7	78.3	77.3	78.7			
*k*_3_ (Sensory quality)	69.7	78.0	76.0	76.3			
*R* (Hardness)	340	59.0	113	95.0			
*R* (Brittleness)	112	32.3	18.3	45.3			
*R* (Sensory scores)	16.0	4.3	1.33	3.33			

^†^ Data are expressed as mean ± standard deviation (*n* = 6). Values with different superscript letters within a row are significantly different (*p* < .05).

The sensory scores of the cookies changed with increasing mushroom flour content (Figure 2a). Initially, the sensory scores of the cookies improved when the level of mushroom flour was increased from 0% to 15%, which was mainly attributed to the desirable flavors associated with the *P. eryngii*. However, as shown in Table 5, adding more than 15% of the mushroom flour had an adverse effect on the sensory scores (texture and flavor score) of the cookies, which was attributed to the change in texture due to the weakening of the gluten network in the dough.

**Table 5:** Effect of mushroom flour on the quantitative sensory properties of cookies (L_9_(3)^4^ results)

	(A) Addition of *Pleurotus eryngii* (%)	(B) Addition of maltodextrin (%)	(C) Addition of NaHCO_3_ (%)	Color score	Texture score	Flavor score	Appearance score
1	1 (10%)	1 (5%)	1 (1%)	17 ± 0	17 ± 1	18 ± 1	19 ± 1
2	1	2 (10%)	2 (1.5%)	19 ± 1	20 ± 1	20 ± 0	20 ± 1
3	1	3 (15%)	3 (2%)	18 ± 1	19 ± 0	19 ± 1	19 ± 1
4	2 (15%)	1	2	20 ± 1	21 ± 0	21 ± 1	21 ± 0
5	2	2	3	20 ± 1	22 ± 1	22 ± 2	21 ± 1
6	2	3	1	21 ± 1	23 ± 0	23 ± 1	22 ± 1
7	3 (20%)	1	3	16 ± 2	17 ± 1	17 ± 1	19 ± 1
8	3	2	1	18 ± 1	18 ± 1	18 ± 0	17 ± 1
9	3	3	2	18 ± 1	17 ± 1	18 ± 0	17 ± 1

In summary, considering the influence of all three properties of the cookies (hardness, brittleness, and sensory scores), the most significant factor influencing the quality of cookies was A (mushroom flour), followed by B (maltodextrin), and then C (NaHCO_3_). The maximum sensory evaluation scores of the cookies were obtained when mushroom flour addition, maltodextrin addition, and NaHCO_3_ addition were A2B2C2 (15% mushroom flour, 10% maltodextrin, and 1.5% sodium bicarbonate) according to the sensory evaluation scores. There were no significant differences in the sensory evaluation scores of A2B2C2 with the best combination on the orthogonal test table (A2B3C1) through validation tests. However, taking into account economic factors of factory production and the trend toward low‐carbohydrate foods, A2B2C2 (15% *P. eryngii* flour, 10% maltodextrin, and 1.5% NaHCO_3_) was selected as the best cookie formulation because of the lower maltodextrin content and the cookies formulation was confirmed by addition of experiment cookies with the best formulation.

## 4 CONCLUSIONS

The addition of wheat flour with mushroom (*P. eryngii*) flour influenced the mixing and rheological properties of the blended dough. The water absorption capacity and peak viscosity of mixed flours significantly increased when more than 15% mushroom flour was incorporated. The dough stability time decreased when the ratio of mushroom flour increased. The supplementation of mushroom flour lowered the gelatinization temperature and the shear modulus values. Analyses of cookie quality properties indicated that fortification with 15% mushroom flour was the maximum level of addition recommended for a better dough functionality and acceptable cookie quality. An orthogonal test showed that A2B2C2 (15% mushroom flour, 10% maltodextrin, and 1.5% sodium bicarbonate) was the best combination for producing healthier cookies with good taste attributes.

## CONFLICT OF INTEREST

The authors declare no conflict of interest.

## ETHICAL APPROVAL

This study does not involve any human or animal testing.
